# Novel Application of Fluorescence Lifetime and Fluorescence Microscopy Enables Quantitative Access to Subcellular Dynamics in Plant Cells

**DOI:** 10.1371/journal.pone.0005716

**Published:** 2009-05-27

**Authors:** Kirstin Elgass, Katharina Caesar, Frank Schleifenbaum, York-Dieter Stierhof, Alfred J. Meixner, Klaus Harter

**Affiliations:** 1 Institute for Physical and Theoretical Chemistry, University of Tübingen, Tübingen, Germany; 2 Center for Plant Molecular Biology, Department of Plant Physiology, University of Tübingen, Tübingen, Germany; 3 Center for Plant Molecular Biology, Microscopy, University of Tübingen, Tübingen, Germany; Umeå Plant Science Centre, Sweden

## Abstract

**Background:**

Optical and spectroscopic technologies working at subcellular resolution with quantitative output are required for a deeper understanding of molecular processes and mechanisms in living cells. Such technologies are prerequisite for the realisation of predictive biology at cellular and subcellular level. However, although established in the physical sciences, these techniques are rarely applied to cell biology in the plant sciences.

**Principal Findings:**

Here, we present a combined application of one-chromophore fluorescence lifetime microscopy and wavelength-selective fluorescence microscopy to analyse the function of a GFP fusion of the Brassinosteroid Insensitive 1 Receptor (BRI1-GFP) with high spatial and temporal resolution in living *Arabidopsis* cells in their tissue environment. We show a rapid, brassinolide-induced cell wall expansion and a fast BR-regulated change in the BRI1-GFP fluorescence lifetime in the plasmamembrane *in vivo*. Both cell wall expansion and changes in fluorescence lifetime reflect early BR-induced and BRI1-dependent physiological or signalling processes. Our experiments also show the potential of one-chromophore fluorescence lifetime microscopy for the *in vivo* monitoring of the biochemical and biophysical subcellular environment using GFP fusion proteins as probes.

**Significance:**

One-chromophore fluorescence lifetime microscopy, combined with wavelength-specific fluorescence microscopy, opens up new frontiers for *in vivo* dynamic and quantitative analysis of cellular processes at high resolution which are not addressable by pure imaging technologies or transmission electron microscopy.

## Introduction

Fluorescence microscopy is a powerful tool for studying the subcellular partitioning and intracellular dynamics of fluorophor-tagged proteins in living cells [1]–[3]. It has become possible to combine fluorescence microscopy with new time-resolved laser spectroscopic methods. Higher-order pulsed laser irradiation (Stimulated Emission Depletion microscopy, STED) [4]–[5] or single molecule blinking statistics (PhotoActivated Localization Microscopy, PALM) [6] and high resolution colocalization of single molecules (STORM) [7] are used for obtaining ultra-high spatial resolution far below the diffraction limit. For observing dynamic processes associated with single molecules, techniques such as fluorescence correlation spectroscopy (FCS) are available [8]–[13]. However, the high resolution and sensitive fluorescence microscopy of proteins, such as membrane-bound receptor kinases, is difficult to carry out in living plant cells, especially in the context of their tissue, due to the autofluorescence of cellular compartments such as the cell wall. This problem is usually circumvented by studying proteins of higher plants in mammalian cells [14], plant protoplasts [15]–[16] or plant cells after plasmolysis [17]. An *in vivo* FCS analysis of phytochrome mobility was reported for moss protonemal tip cells [18]. However, protoplast preparation and plasmolysis induce stress responses and may modify the subcellular partitioning, intracellular dynamics and activity of the receptors. Furthermore, expression of plant receptors in heterologous systems may lead to artificial results. Due to these limitations, our knowledge on cell biological mechanisms, by which, for instance, membrane-associated receptors act at the plant cell surface, traffic inside the plant cell and induce early cellular responses, is restricted.

To overcome these limitations, we applied one-chromophore fluorescence lifetime microscopy (ocFLM), combined with wavelength-selective fluorescence microscopy, to plant cells in their tissue environment in *Arabidopsis thaliana* at high local resolution. Our ocFLM system consisted of a confocal sample scanning microscope (CSSM), a spectrally integrating detector for measuring fluorescence intensity and a time-correlated single-photon counting board for recording fluorescence lifetime decays, which was custom-built in our lab [19]–[20]. For plant material, light-grown seedlings expressing a BRI1-GFP fusion protein were used. BRI1, a plasmalemma-bound receptor kinase for plant steroids like brassinolide (BL) [21], recycles through endosomes [22]–[23] and regulates many aspects of growth and differentiation, including cell expansion [24].

As shown here, our combined optical-spectroscopic application, together with a novel data analysis approach using BRI1-GFP as a model receptor, provides unprecedented insight into cells and opens up new frontiers for *in vivo* dynamic, quantitative analysis of molecular and subcellular processes at high local resolution.

## Results

### Differentiation of GFP and fluorescence background

**Figure 1:**
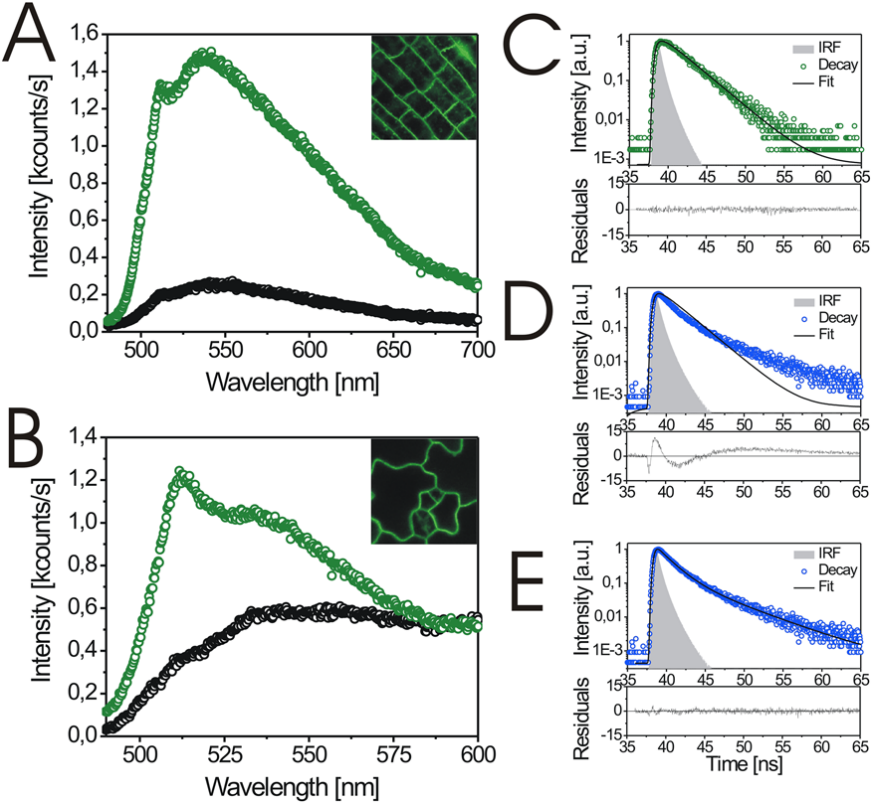
Differentiation of autofluorescence and the GFP signal in BRI1-GFP-expressing and wildtype *Arabidopsis* cells. (A–B) Wavelength-resolved fluorescence spectra from plasmalemma-cell wall sections of BRI1-GFP-expressing cells (green) and wildtype cells (black). The spectra were recorded in root, A, or cotyledon cells, B. The areas (50×50 µm) used for recording are shown in the inlets. (C) *In vivo* decay trace of fluorescence lifetime from BRI1-GFP expressing hypocotyl cells fitted by a mono-exponential function (black line). The lifetime decay was measured at the peak of fluorescence intensity at 500 to 512 nm. (D) *In vivo* decay trace of autofluorescence lifetime in the plasmalemma-cell wall area of wildtype hypocotyl cells fitted by a mono-exponential function (black line). (E) *In vivo* decay trace of autofluorescence lifetime in the plasmalemma-cell wall area of wildtype hypocotyl cells fitted by a multi-exponential function (black line). The lifetime decay was measured at around 500 to 512 nm. The residuals indicate the deviation between the measured and the model decay function. In a good fit the residuals are distributed symmetrically around 0. IRF, instrument response function.

To differentiate between background fluorescence and the BRI1-GFP signal, we recorded a two-dimensional fluorescence intensity image over the plasmalemma-cell wall area of BRI1-GFP-expressing and wild type seedlings in root and shoot (cotyledon) cells. In contrast to the spectra of non-transformed wild type cells (Fig. 1A and B, black dots), the spectra of the BRI1-GFP-expressing cells showed a higher overall intensity and two main peaks (Fig. 1A and B, green dots). These two peaks are typical of and specific for GFP in aqueous solution (Figure S1) [25]. However, the spectra recorded from the plants differed significantly and the shoulder amplitudes and intensity ratios of the two maxima varied between root and cotyledon cells (Fig. 1A and B). These data indicate that the degree and properties of autofluorescence depend on the cell type and the autofluorescence strongly interferes with the spectrum of GFP fluorescence emission. Secondly, we recorded fluorescence lifetime decay traces from a selected probe volume with pulsed laser excitation in living plant cells. At the maximum of fluorescence emission between 500 and 512 nm of BRI1-GFP expressing hypocotyl cells (Fig. 1B), the lifetime was characterized by a mono-exponential decay (Fig. 1C) typical for GFP (Figure S1) [26]–[27]. In contrast, the background autofluorescence, which was recorded at the identical wavelength range in wildtype hypocotyl cells, strongly deviated from the mono-exponential decay function (Fig. 1D). An acceptable fit to the experimental data with respect to the average lifetime and amplitude could only be achieved by a multi-exponential decay function (Fig. 1E, see Table S1 for additional information on the fitting parameters of all lifetime decay traces shown here).

**Figure 2:**
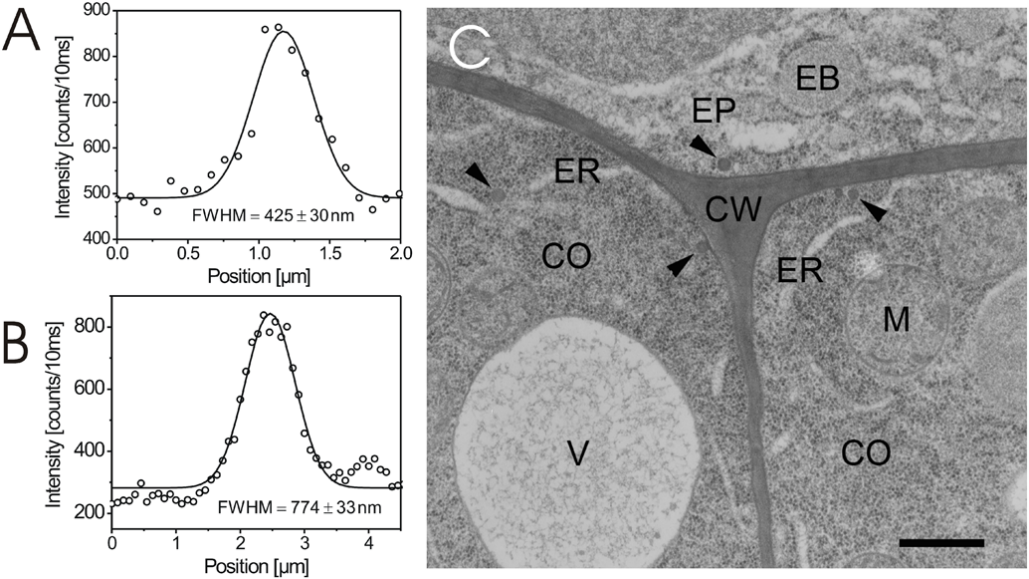
Dimensional appearance of periclinal and anticlinal cell walls in confocal and TEM images. (A) Fluorescence intensity curve recorded over a plasmalemmata-wall section of the periclinal wall of BRI1-GFP expressing *Arabidopsis* root cells. The full width at half maximum value (FWHM) of the Gaussian fitting revealed an apparent wall thickness of 425±30 nm. (B) Fluorescence intensity curve recorded over a plasmalemmata-wall section of the anticlinal wall of the identical root cell described in A. The FWHM of the Gaussian fitting revealed an apparent wall thickness of 773±33 nm. (C) Ultrathin TEM image of a tissue section cutting three different root cells. Two anticlinal and one periclinal cell walls (CW) are shown. CO, cortical cell; EP, epidermal cell; ER, endoplasmatic reticulum; EB, ER body; M, mitochondrium; V, vacuole. The arrow heads point to Golgi-derived vesicles. The bar represents 500 nm.

Furthermore, we were faced with the problem that the cell wall in the CSSM images appeared to be much thicker than when it was determined by transmission electron microscopy (TEM) (Fig. 2). However, one has to take into account the non-point-shaped dimension of the confocal laser beam focus. When this is considered, the measured full width at half maximum (FWHM) values of the Gaussian fits of the fluorescence intensity distribution of 425 nm for the periclinal cell wall (Fig. 2A) and 774 nm for the anticlinal cell wall (Fig. 2B) reflect a thickness of 83 nm and 432 nm, respectively (see Text S1 for calculation). The values of the periclinal cell walls are, therefore, consistent with those determined by TEM (about 45 to 100 nm; Fig. 2C; Table S5). The width of the anticlinal walls detected by our fluorescence intensity approach was still too large to correspond to our TEM measurements. However, to understand the values determined for the longer anticlinal walls, one has to take into account that a cell wall slanted to a few degrees appears to have a higher CSSM-measured thickness. For instance, imaging cell membranes bordering a cell wall with a real thickness of 100 nm at a slant of 30° generates a FWHM value of around 730 nm (see Text S1 for calculation). Thus, the prevailing larger FWHM values of the anticlinal cell wall measurements can be explained by their higher bending probability which results in higher slanting effects in the focus of the confocal microscope. Therefore, for an accurate determination of small changes in the expansion of plasmamembrane-cell wall areas, the selected section has to be kept in tight focus during the entire analysis, which is enabled by our experimental CSSM setup.

**Figure 3:**
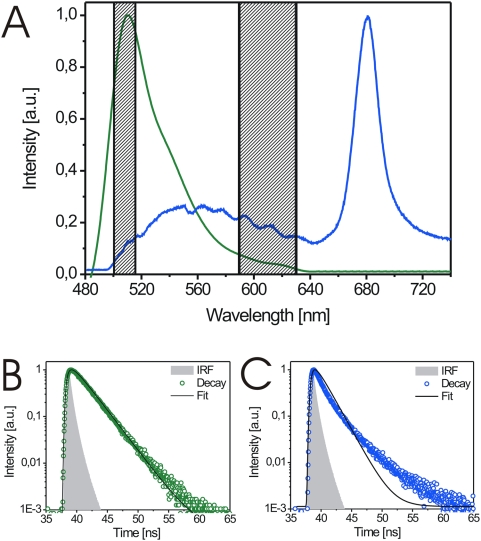
Measurement ranges for comparison between GFP-fluorescence and cell wall-derived autofluorescence. (A) Overlap of the fluorescence spectrum of purified GFP in water (green) and the autofluorescence spectrum (blue) of wildtype *Arabidopsis* hypocotyl cells. The broad band of the spectrum (500–640 nm, blue) originates from the cell wall, the sharp peak at 680 nm originates from chlorophyll. The grey hatched areas show the spectral ranges used for recording GFP-fluorescence (around 500 nm) and autofluorescence, respectively (around 600 nm). (B) Fluorescence decay trace recorded in the 500 nm region (left hatched bar in A) in a BRI1-GFP expressing hypocotyl cell, fitted by a mono-exponential function (black line), proves the main presence of GFP-fluorescence. (C) Fluorescence decay trace recorded in the 600 nm region (right hatched bar in A) in a BRI1-GFP expressing hypocotyl cell, fitted by a mono-exponential function (black line), proves the dominant presence of autofluorescence. IRF, instrument response function.

In addition, the differences of GFP and background fluorescence with respect to their spectra and fluorescence lifetime enabled us to choose the appropriate experimental set-up for the distinction of subcellular compartments such as the plasmalemma and the cell wall (Fig. 3). Thus, we used the emission range at around 500 nm for recording GFP fluorescence and the range at around 600 nm for recording cell wall autofluorescence (hatched bars in Fig. 3A).

### Rapid BL-induced cell wall expansion *in vivo*

**Figure 4:**
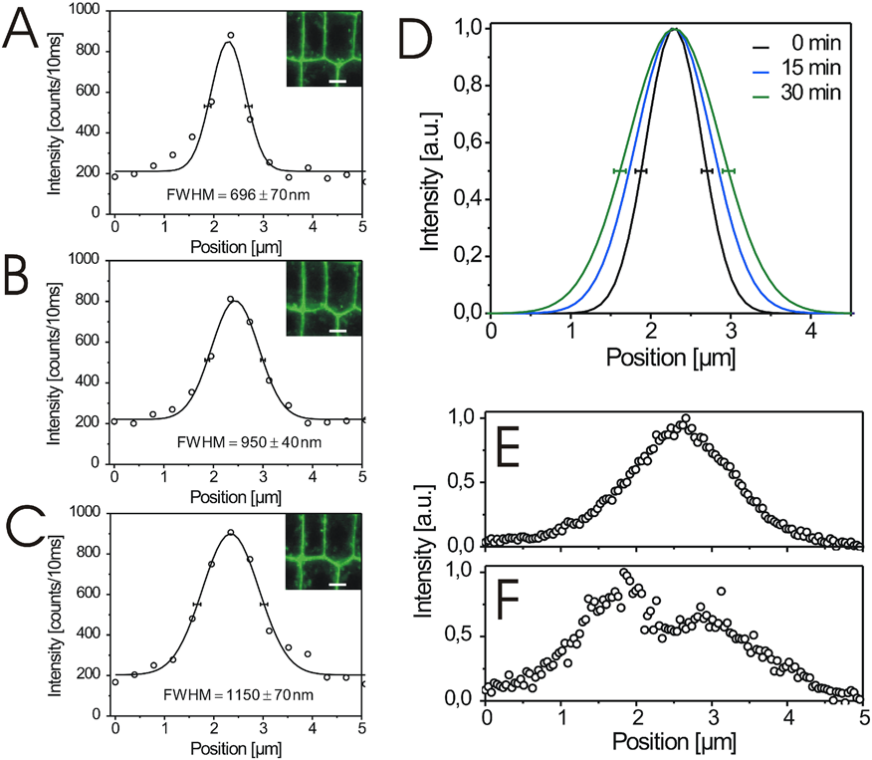
BL-induced expansion of the GFP fluorescence in BRI1-GFP expressing root tips and hypocotyl cells. (A–C) Fluorescence intensity images and the corresponding profiles (Gaussian fits including FWHM values) of root tip cells recorded over the subcellular section indicated by the white line (representing 5 µm) shown in the confocal image inlets before (0 min) A, 15 min, B, and 30 min, C, after addition of 10 nM BL. (D) Combined Gaussian fits of the intensity profiles shown in A–C. (E–F) Fluorescence intensity profiles recorded over a 5 µm section of the plasmalemmata-cell wall area of two neighbouring hypocotyl cells before, E, and 30 min after application of 10 nM BL, F. For the determination of the FWHM error see Material and Methods. For the statistical analysis of the BRI1-GFP fluorescence measurements see Table S2.

**Figure 5:**
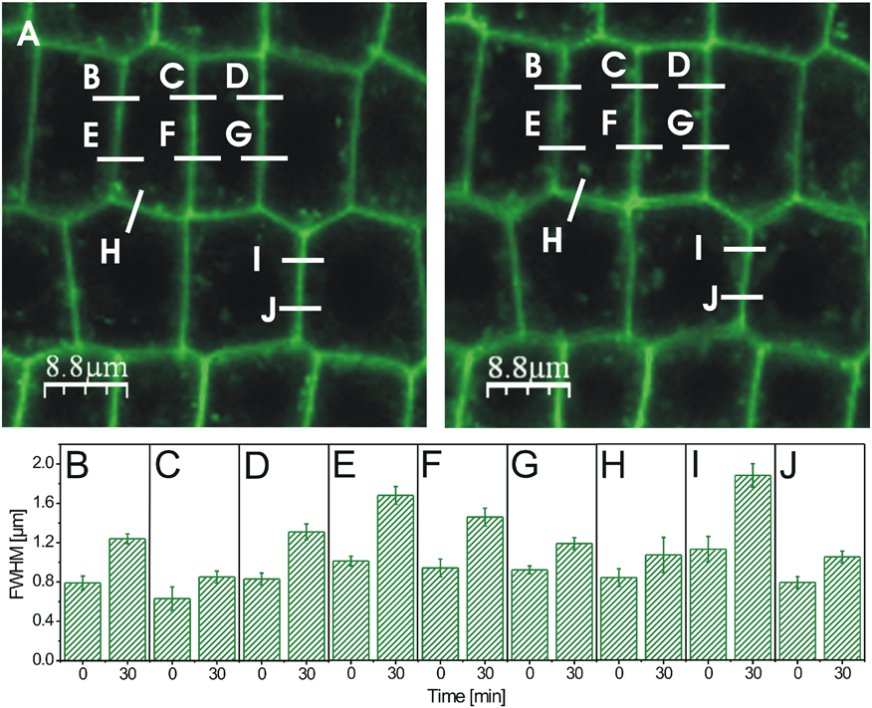
BL-induced expansion of the GFP fluorescence signal in BRI1-GFP expressing root tip cells. (A) Confocal image of root tip tissue before (left) and 30 min after addition of 10 nM BL (right). Fluorescence intensity curves were recorded over the plasmalemmata-cell wall sections indicated by the white, alphabetically numbered lines and the FWHM values of their Gaussian fits calculated before and 30 min after addition of BL. (B–J) FWHM values of the plasmalemmata-cell wall sections indicated in A before (0 min) and 30 min after addition of 10 nM BL. For the determination of the FWHM error in B to J see Material and Methods. For the statistical analysis of the BRI1-GFP fluorescence measurements see Table S2.

We next addressed the problem of real-time measurement of possible physiological effects of BRI1-GFP at subcellular level in living plant cells. Former studies have shown that BL does not alter the fluorescence intensity, the number of vesicles in the endosomal pool and the intracellular distribution of BRI1-GFP in root cells [22]. This is in agreement with our observations (Fig. 4A to C, Fig. 5 and data not shown). However, the local fluorescence intensity of BRI1-GFP as a function of BL, time and a defined subcellular area was not yet studied. We therefore, recorded fluorescence intensity profiles over selected plasmalemmata-cell wall sections before and after treating the cells for 15 to 30 min with 10 nM BL. The intensity profiles enabled us to discriminate between the fluorescence signal of the proper plasmalemmata-cell wall section and membrane vesicles, which budded from the plasmalemmata during the incubation time. As shown in Fig. 4A–D and Fig. 5, the treatment of seedlings with BL induced an expansion of the BRI1-GFP signal in the plasmalemma-cell wall section of the root cells within 30 min. This response was also observed in hypocotyl cells, where BL induced an expansion of the BRI1-GFP signal of two neighboring cells, so that the plasmalemmata became optically distinguishable (Fig. 4E and F). The degree of expansion did not only depend on the cell type but also on the position of the measured section along the cell wall of individual cells (Fig. 5). The Gaussian fitting of the GFP intensity profiles of more than 30 independent sections measured in several individual cells from 5 seedlings revealed that the fluorescence signals expanded around 34±22% (n = 31, p = 0,0003) in response to BL treatment (see Table S2 for the single measurement values).

**Figure 6:**
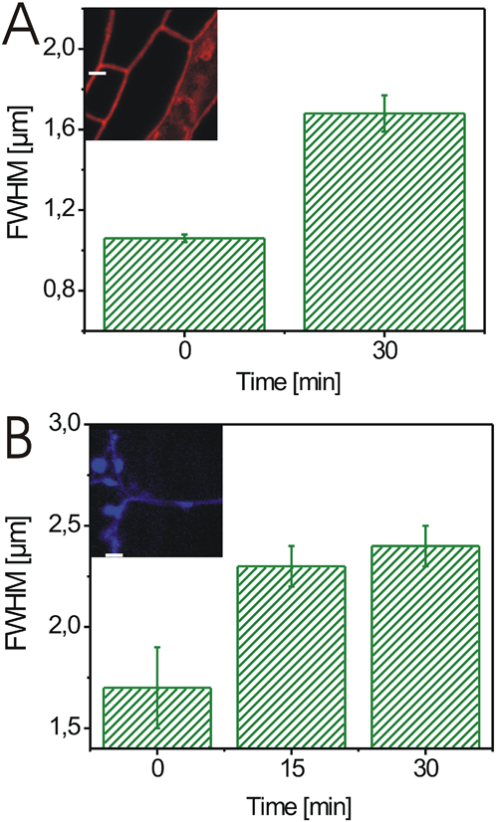
BL-induced expansion of the cell wall in BRI1-GFP expressing hypocotyl cells. (A) FWHM values of the anticlinal wall autofluorescence from hypocotyl cells recorded over the subcellular section indicated by the white line (10 µm) shown in the confocal image inlet at 0 min and 30 min after addition of 10 nM BL. (B) FWHM values of Calcofluor-stained anticlinal wall from hypocotyl cells recorded over the subcellular section indicated by the white line (11 µm) shown in the confocal image inlet at 0, 15 and 30 min after addition of 10 nM BL. For the determination of the FWHM error in A and B see Material and Methods. For the statistical analysis of the cell wall autofluorescence and Calcofluor fluorescence measurements see Tables S3 and S4.

The spatial separation of the plasmalemmata could have been caused by BL-induced plasmolysis or BL-induced cell wall expansion. Therefore, we determined the spatial behavior of the cell wall in response to BL treatment in BRI1-GFP-expressing seedlings. The measurement of fluorescence from different subcellular origins was possible as demonstrated before (Fig. 3). The cell wall autofluorescence expanded around 41±24% (n = 29, p = 0,0145) after brassinolide treatment coincidentally with the separation of the plasmalemmata in the analysed cells (see Table S3 for the single measurement values and Fig. 6A as an example). A BL-induced expansion of cell wall by 49±29% (n = 15, p = 0,0131) was also observed when the BRI1-GFP expressing seedlings were stained with the cell wall-staining dye Calcofluor (Fig. 6B, see Table S4 for the single measurement values).

**Figure 7:**
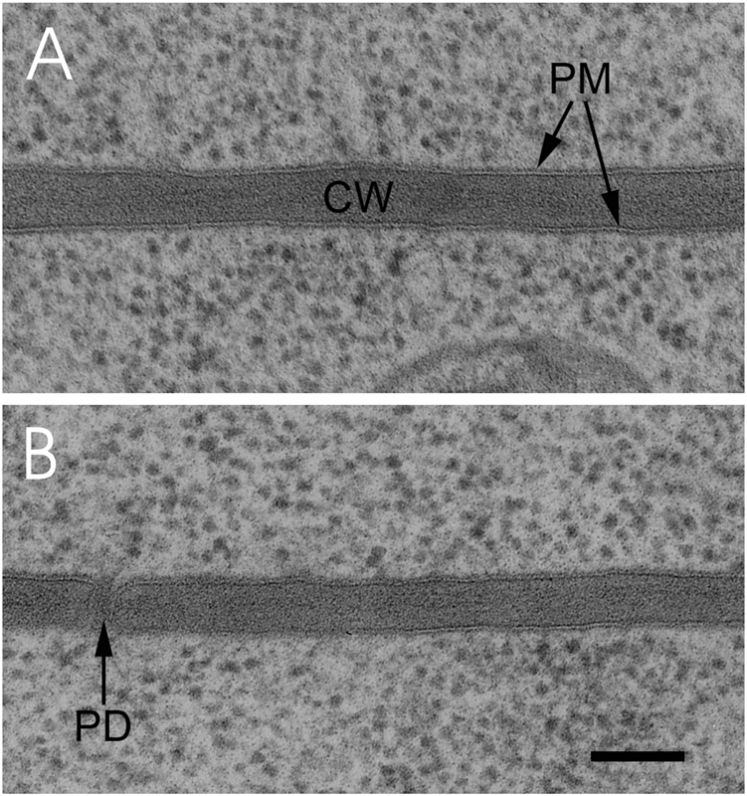
TEM images of ultrathin sections from different root cells of BRI1-GFP expressing seedlings. (A) Ultrathin section of a root cell from a seedling high-pressure frozen 30 min after the addition of 10 nM BL. (B) Ultrathin section of an independent root cell from a mock-treated, high-pressure frozen seedling. The cell walls of epidermal cells (upper cells) facing cortex cells (lower cell) are shown. PM, plasmalemma; CW, cell wall; PD, plasmadesmon. The bar represents 100 nm. For the statistical analysis of the cell wall measurements see Table S5.

We also carried out a TEM analysis of control and BL-treated (30 min) root cells. Root tips from two seedlings of each were frozen under high pressure, freeze-substituted in osmium tetroxide containing acetone, embedded in epoxy resin, cut and subjected to TEM. Using the TEM images, the width of 72 periclinal cell walls of each was measured, and the mean width calculated. The measurements resulted in a wall width of 62,5±10,8 nm for the control and 64,9±10,1 nm for BL-treated root tip cells (Fig. 7; see Table S 5 for the single measurement values). Thus, a statistically significant difference with high confidence between BL-treated and mock-treated seedlings (p = 0,0845) could not be observed by using TEM. We realized, however, that the variation of the wall width between individual cells and within an individual cell was high and ranged from 47 to 88 nm in the mock-treated and from 49 to 94 nm in the BL-treated root tips (Table S 5; see Discussion for the set of difficulties with respect to the TEM measurements). In addition, our data showed that the cellular structures including the plasmalemmata and cell walls were normal in BL-treated as well as in untreated cells (Figs. 2 and 7). Furthermore, there was no detachment of the plasmalemmata from the cell wall in response to BL treatment (Fig. 7). The latter observation excludes a possible plasmolytic cause for the BL-induced expansion in the BRI1-GFP signal.

**Figure 8:**
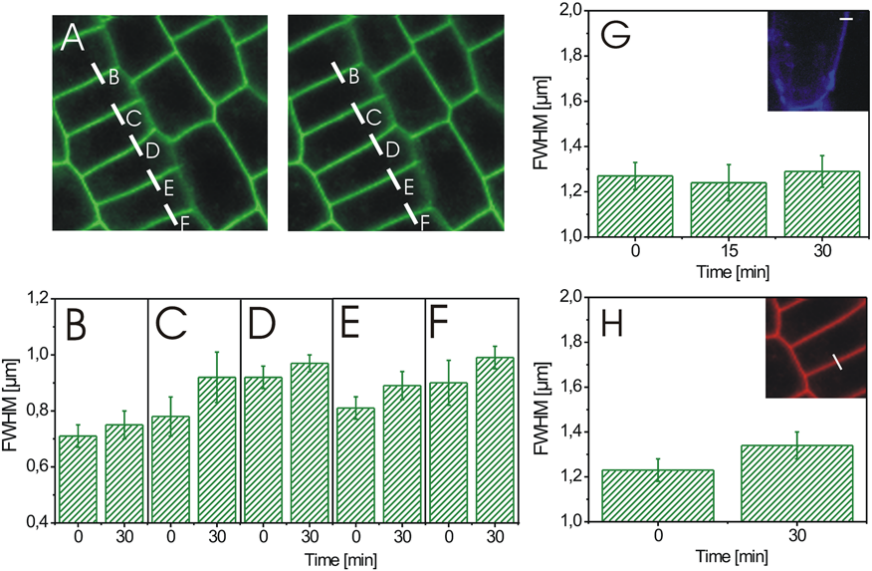
BL-induced changes in the cell wall width of aquaporin-GFP expressing *Arabidopsis* cells. (A) Confocal image of root tip tissue before (left) and 30 min after addition of 10 nM BL (right). Fluorescence intensity curves were recorded over the plasmalemmata-cell wall sections indicated by the white, alphabetically numbered lines and the FWHM values of their Gaussian fits calculated. (B–F) FWHM values of the plasmalemmata-cell wall sections indicated in A before (0 min) and 30 min after addition of 10 nM BL. (G) FWHM values of Calcofluor-stained anticlinal wall from hypocotyl cells recorded over the subcellular section indicated by the white line (8 µm) shown in the confocal image inlet at 0, 15 and 30 min after addition of 10 nM brassinolide. (H) FWHM values of the anticlinal wall autofluorescence from hypocotyl cells recorded over the subcellular section indicated by the white line (5 µm) shown in the confocal image inlet at 0 min and 30 min after addition of 10 nM BL. For the determination of the FWHM error in B to H see Material and Methods. For the statistical analysis of the aquaporin-GFP fluorescence measurements see Table S6.

To determine the specificity of the response, we carried out identical experiments on seedlings, expressing plasmalemma-located aquaporin-GFP [28] and wildtype seedlings. Our experiments revealed a slight BL-induced separation of adjacent GFP signals by 10±8% (see Table S6 for statistics and single measurement values; Fig. 8A–F) in aquaporin-GFP expressing seedlings and a slight expansion of the cell wall autofluorescence by 18±16% in wild type seedlings (see Table S7 for statistics and single measurement values) without general statistical significance. We obtained similar results, when Calcofluor-stained cell walls or the autofluorescence of the cell walls in aquaporin-GFP-expressing cells were measured (Fig. 8G–H; data not shown).

### Monitoring the environment of plasmalemma-bound GFP fusion proteins *in vivo*

**Figure 9:**
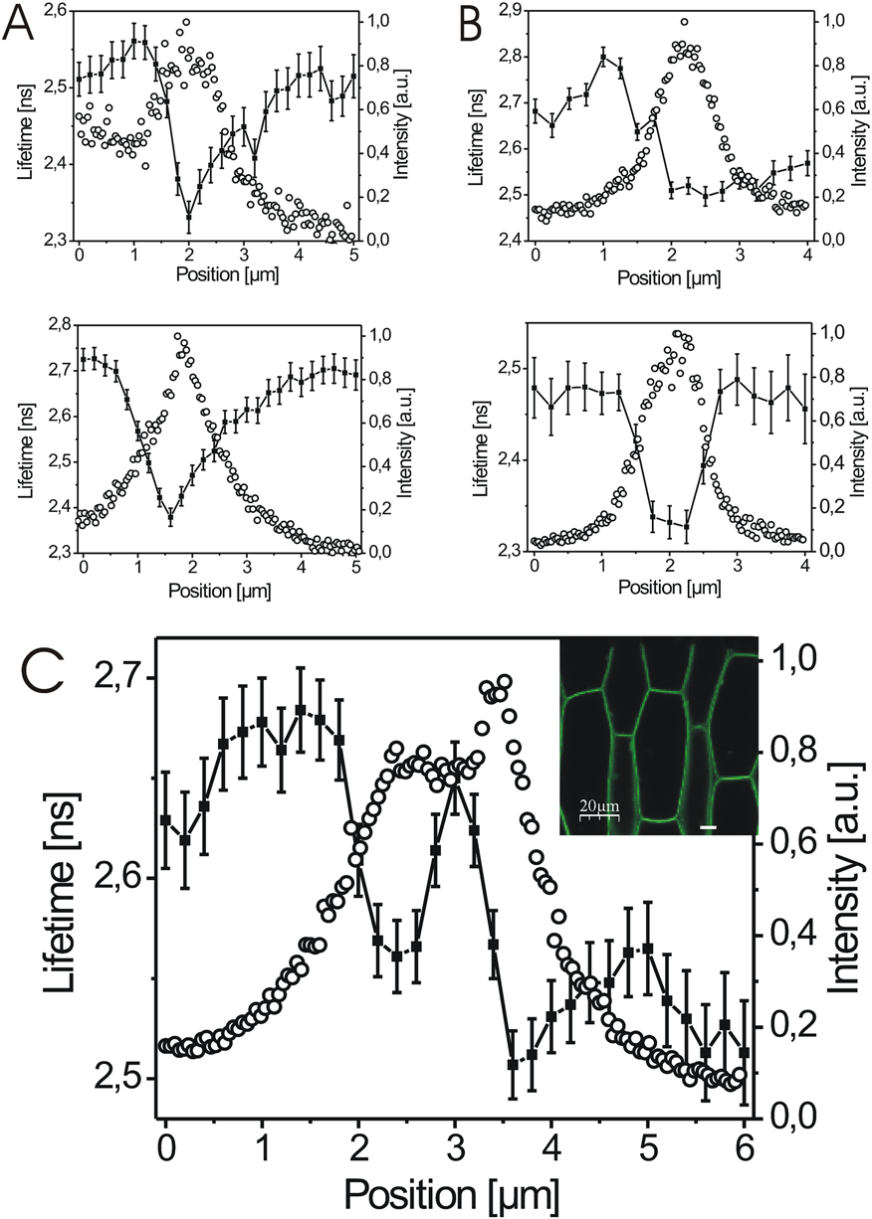
ocFLM of BRI1-GFP and aquaporin-GFP reveals gradients in their subcellular environment. (A) GFP fluorescence lifetimes (filled squares) and the corresponding intensity profiles (open circles) recorded over 5.0 µm plasmalemma-cell wall sections of two different hypocotyl cells from two independent BRI1-GFP expressing *Arabidopsis* seedlings. (B) GFP fluorescence lifetimes (filled squares) and the corresponding intensity profiles (open circles) recorded over 4.0 µm plasmalemma-cell wall sections of two different hypocotyl cells from two independent aquaporin-GFP expressing *Arabidopsis* seedlings. (C) GFP fluorescence lifetime (filled squares) and the corresponding intensity profile (open circles) over a 6.0 µm plasmalemma-cell wall area of a hypocotyl cell from a BRI1-GFP expressing *Arabidopsis* seedling. The white line in the confocal image inlet shows the recorded section. For the calculation of the fluorescence lifetime values and error bars see Material and Methods. Additional BRI1-GFP and aquaporin-GFP fluorescence lifetime measurements are presented in Fig. 10D and E.

The lifetime of GFP fluorescence provides information about the physical and chemical environment of the fluorophore and, thus, the GFP fusion protein [29]–[30]. In addition to fluorescence intensity, we recorded GFP fluorescence lifetime decay traces across plasmalemmata-cell wall sections in hypocotyl cells of aquaporin-GFP and BRI1-GFP expressing seedlings at up to 200 nm intervals. The measurements were based on a different but sufficient number of fluorescence counts (Table S1), which is reflected in the error calculation [31]. We observed significant differences in GFP fluorescence lifetime across plasmalemma-cell wall sections and regularly also between adjacent cells for both aquaporin-GFP and BRI1-GFP (Fig. 9A and B). A detailed subcellular analysis revealed that the most significant differences in BRI1-GFP lifetime existed within the cell between the cytoplasm, plasmalemma and cell wall (Fig. 9C).

**Figure 10:**
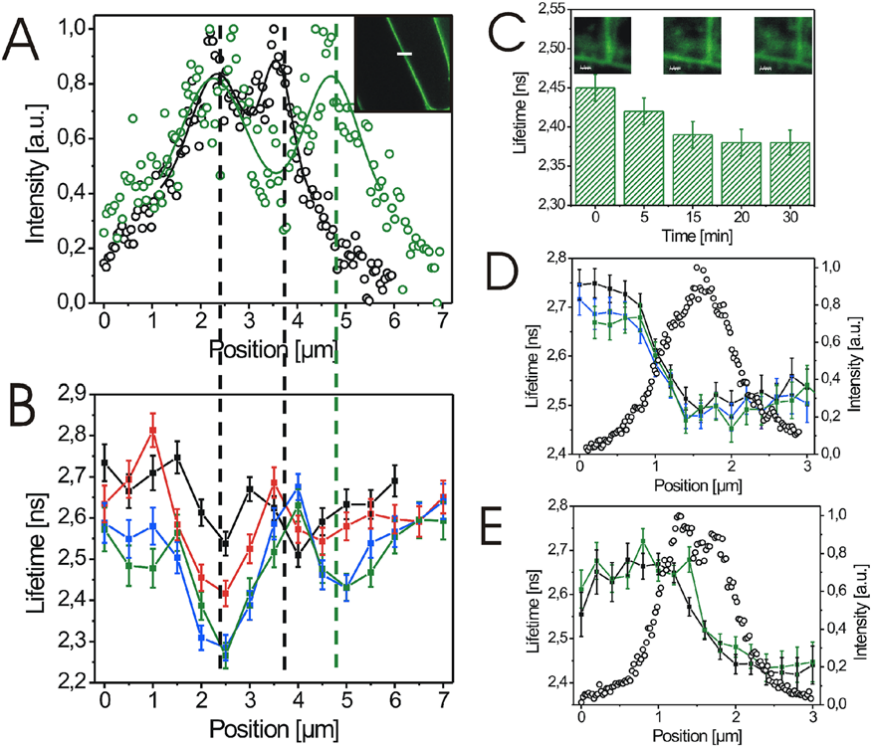
BL-induced cell wall expansion is paralleled by changes in the BRI1-GFP fluorescence lifetime. (A) BL-induced cell wall expansion of BRI1-GFP expressing hypocotyl cells from *Arabidopsis* seedlings before (black circles) and 30 min after (green circles) hormone application. The fluorescence intensity was recorded over a 7.0 µm section as indicated by the white line in the confocal image inlet. (B) Fluorescence lifetimes of BRI1-GFP in the identical plasmalemma-cell wall section shown in A before (black squares), 10 (red squares), 20 (blue squares) and 30 min (green squares) after addition of 25 nM BL. The minima in the lifetime curves correspond to the maxima in the intensity profiles shown in A as indicated by the dashed lines. (C) Fluorescence lifetimes of BRI1-GFP in root tip cells 0 to 30 min after application of 25 nM BL. Lifetimes were obtained by integrating over all recorded pixels of the confocal image. (D) GFP fluorescence lifetime (filled squares) and the corresponding intensity profile (open circles) recorded over a 3.0 µm plasmalemma-cell wall section of a BRI1-GFP expressing hypocotyl cell before (black squares), 15 min (blue squares) and 30 min (green squares) after onset of mock treatment. (E) GFP fluorescence lifetime (filled squares) and the corresponding intensity profile (open circles) recorded over a 3.0 µm plasmalemma-cell wall section of an aquaporin-GFP expressing hypocotyl cell before (black squares) and 30 min (green squares) after application of 25 nM BL. For the calculation of the lifetime values and error bars see Material and Methods. The experiments in B and C were repeated three times using cells from three independent seedlings. Representative results are shown. The results of the additional BRI1-GFP lifetime measurements along plasmalemma-cell wall sections are presented in Figure S2.

To determine whether BL also induces changes in the environment of BRI1-GFP, we analysed the fluorescence lifetime of the receptor fusion. After the addition of BL, we again observed cell wall expansion (Fig. 10A). Furthermore, BRI1-GFP fluorescence lifetime at the plasmalemmata strongly decreased within 20 min of BL addition and then remained constant (Fig. 10B, C; Figure S2). In contrast, fluorescence lifetime in the subcellular areas adjacent to the plasmalemma region remained largely unchanged (Fig. 10B). By the spatial extension of our measurements, BL-induced changes in BRI1-GFP lifetime were not only observed in sections over the plasmalemma-wall areas but also over larger cell areas (Fig. 10C). Significant changes in GFP fluorescence lifetime were never measured after mock-treatment of the BRI1-GFP line or in the BL-treated aquaporin-GFP expressing cells (Fig. 10D and E; data not shown).

### BR-induced responses are cell physiological processes

**Figure 11:**
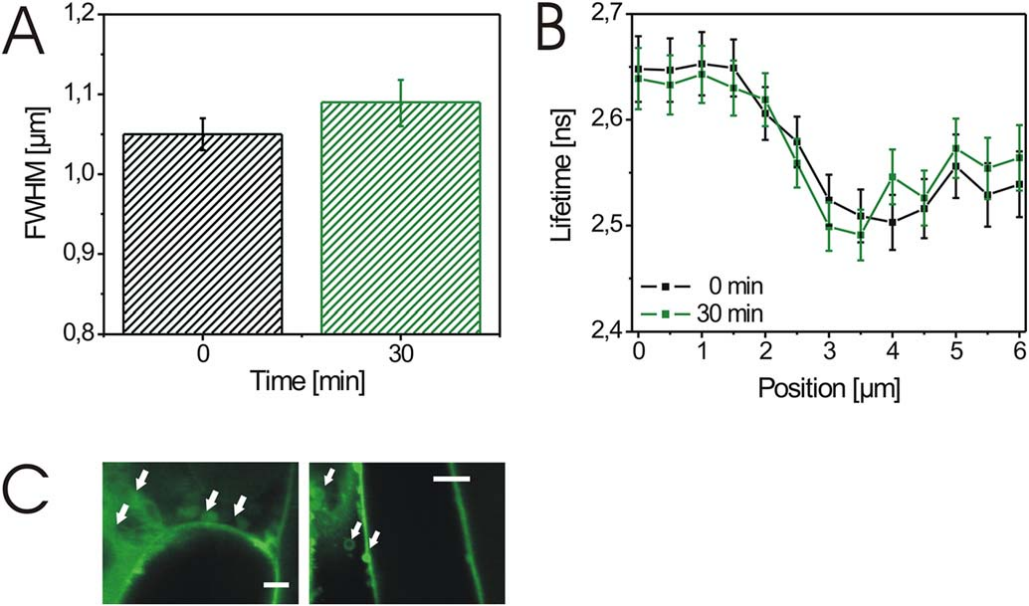
BL-induced wall expansion and changes in BRI1-GFP fluorescence lifetime require a functional intracellular trafficking system. (A) FWHM values of GFP intensity profiles recorded over a 4.0 µm plasmalemmata-cell wall section of hypocotyl cells from BRI1-GFP expressing *Arabidopsis* seedlings in the presence of 50 µM BFA before (0 min) and 30 min after application of 10 nM BL. For the determination of the FWHM error see Material and Methods. (B) Fluorescence lifetimes of BRI1-GFP in the identical plasmalemma-cell wall section shown in A in the presence of 50 µM BFA before (black squares) and 30 min (green squares) after addition of 25 nM BL. For the calculation of the lifetime values and error see Material and Methods. (C) Confocal images of hypocotyl cells treated with 50 µM BFA. The BFA compartments are indicated by white arrows. The white bars represent 6 µm (left) and 10 µm (right). The experiments in A and B were repeated four times using cells from four independent seedlings. One representative result is presented. The results of additional measurements are shown in Figure S3.

Recent studies suggested that the endosomal pool of BRI1 is critical for the signaling and regulation of BL-responsive genes in *Arabidopsis* as shown by treatment with Brefeldin A (BFA) [22]–[23]. BFA inhibits the function of ARF-GTPases by interacting with their associated GEFs and, thus, has strong effects on the integrity of subcellular compartments and the endosomal vesicle pool by inhibiting intracellular trafficking pathways [32]–[33]. We, therefore, addressed the question whether BFA treatment also interferes with BL-induced cell wall expansion and BRI-GFP fluorescence lifetime. In the presence of the inhibitor BFA compartments appeared and both processes were strongly inhibited, suggesting that an intact intracellular trafficking system is required for their execution (Fig. 11 and Figure S3).

## Discussion

### Specific GFP versus unspecific compartment fluorescence

As shown in Figure 1, there is a significant, cell-type specific background fluorescence signal which mainly derives from the wall of the plant cell. This unspecific background fluorescence can be differentiated from the GFP signal by its spectra and also by its characteristic fluorescence decay pattern. Whereas GFP fluorescence shows a prototypical monoexponential decay function, background fluorescence can only be described by a multiexponential decay function. This suggests that the unspecific background fluorescence at the excitation wavelength of 473 nm originates from different components inside the cell wall, which have different excited-state lifetimes. Thus, the combination of wavelength- and time-resolved fluorescence spectroscopy enables us to distinguish unambiguously between a specific signal and background fluorescence in a cell type-specific manner, dispensing with the use of additional fluorescent dyes, which might interfere with cellular processes. These differences in lifetime also allowed us to establish a procedure for the efficient background correction and, thus, the improvement of the signal-to-background ratio in living plant cells (patent application no. 10 2009 005 953 from 19.01.09; F. Schleifenbaum, K. Elgass, M. Sackrow, K. Caesar, K. Berendzen, K. Harter, A. Meixner, manuscript in preparation)

### Measurement of cell wall extension at subcellular resolution

Our data demonstrate that the cell walls significantly expand within a few minutes of BL treatment in BRI1-GFP expressing cells. A slight expansion response was also observed in aquaporin-GFP expressing and wildtype seedlings which was without clear overall statistical significance. However, when determined at single cell level, almost every cell showed a BL-induced wall expansion. This suggests that BRI1-GFP is necessary for the rapid BL-induced expansion of the cell wall but its over-expression is required to produce a significant effect. A more detailed analysis of many cells from independent seedlings showed that there is a linear relationship between BRI1-GFP fluorescence intensity and the degree of cell wall expansion (K. Elgass, A. Meixner, K. Harter, unpublished). BL is known to alter the biophysical properties of the cell wall, such as its relaxation *via* BRI1-mediated transcriptional and post-transcriptional activation of cell wall-loosening enzymes [24], [34]. However, such an early BL-responsive spatio-temporal and BL-dependent expansion has not yet been reported in the cell wall sections of living *Arabidopsis* cells.

From the resolution point of view, it should, in principle, also be possible to determine cell wall expansions of the observed dimension by TEM. However, it is not technically possible to produce a series of TEM sections at the identical subcellular region before and after the addition of BL. Thus, different seedlings have to be used for the comparative physiological analysis using TEM. The set of difficulties is documented by our own TEM analysis, which demonstrates a high variability of cell wall thicknesses between individual cells and even individual periclinal walls. The high variability is probably due to the different age of the cells, which have different wall dimensions, and the difficulty to exactly localize the site of the measurement along the wall. For instance, the wall thickness at the edge of a cell is different form the width in the middle. In addition, although we used cryofixation and freeze-substitution for the preparation of the probes, we can not entirely exclude the possibility that this treatment may influence the water status and spatial dimension of the cell wall. On the one hand, we conclude that it is almost impossible to determine weak, dynamic changes in cell wall expansion using a TEM approach. On the other hand, these findings unequivocally demonstrate the potential of our approach for the analysis of the dynamics of subcellular processes *in vivo*.

### Changes in the close environment of membrane-bound GFP fusion proteins

The observed differences in BRI1-GFP and aquaporin-GFP fluorescence lifetime suggest strong gradients of physico-chemical parameters, such as membrane potential, pH-value, osmotic conditions and refraction index [30], within subcelluar compartments of a single cell and between adjacent cells. Thus, membrane-bound GFP fusion proteins have the potential to be highly sensitive probes for determining such parameters at sub-cellular resolution *in vivo*. Ongoing experiments are aimed at discovering which parameters may influence the fluorescence lifetime of BRI1-GFP, aquaporin-GFP and other fluorophore-tagged proteins *in vivo*.

After the addition of BL, we observed a significant change in the fluorescence lifetime of BRI1-GFP with time, which was not detected in BL-treated aquaporin-GFP expressing plants. These results can be interpreted to suggest that the activation of BRI1-GFP by BL could alter the physico-chemical properties of the plasmalemma or plasmalemma/cytoplasm interface, which do not affect the fluorescence lifetime of aquaporin-GFP. The observed changes would then be a cell physiological response initiated by BL-activated BRI1-GFP. This idea is substantiated by the fact that BFA inhibits the BL-induced change in BRI1-GFP fluorescence lifetime. As BL was shown to inhibit inwardly rectifying K^+^ currents in guard cell protoplasts of *Vicia faba* and to increase ATPase activity in Azuki bean epicotyls and maize roots, leading to proton extrusion [24], [34]–[35], it is possible that the observed change in the fluorescence lifetime of BRI1-GFP might reflect a hormone-induced alteration in ion currents and membrane potential in *Arabidopsis* cells which depends on the presence of BRI1-GFP. The causal relationship between BL-induced BRI1-GFP activation, the change in BRI1-GFP fluorescence lifetime and cell wall expansion is currently under elucidation.

### Summary and concluding remarks

Compared to pure imaging techniques with ultra-high spatial resolution, such as STED [4]–[5], PALM [6] and STORM [7], our application of ocFLM in combination with wavelength-selective fluorescence microscopy and a novel method of data analysis allows the recording of the spectroscopic and fluorescence lifetime data of GFP fusion proteins *in vivo*, providing new information about their molecular properties, cell physiological function and subcellular environment. This enables the unambiguous identification and clear intracellular localisation of GFP fusion proteins in plant cells. Furthermore, ligand-induced physiological responses in plant cells and receptor dynamics at subcellular resolution can be quantitatively recorded. The use of autofluorescence as “native” fluorophore avoids the use of additional chromophors or the expression of further markers which might interfere with the plant cell's physiology. This is a marked advantage of our approach over STED, PALM and STORM. In addition, with an image acquisition time of approximately 3 h, PALM and STORM do not allow the fast recording of subcellular processes. We, therefore, propose that high resolution ocFLM combined with wavelength-selective fluorescence microscopy will not only lead to new experimental abilities in plants but also is a valuable novel tool for the accomplishment of cell biology in any cell system.

## Materials and Methods

### Plant material and growth conditions

BRI1-GFP [22] and aquaporin-GFP [28] expressing seedlings were grown for 5 days at 22°C and under a regime of 14 h light and 10 h darkness on agar containing 0.5 MS media. For measurements seedlings were carefully removed from the plates and transferred onto microscopic slides and covered with water. The BL solutions at the indicated concentrations and Calcofluor (1% w/v) were applied to side of the microscopic slide and allowed to diffuse to the seedling sample. For the response of the cell walls in presence of BFA, the seedlings were pre-incubated for 1 h in 50 µM BFA solution.

### Optical and spectroscopic measurements

The measurements were performed with a homemade CSSM, based on a Zeiss Axiovert, and equipped with a pulsed 473 nm diode laser operating at a repetition rate of 10 MHz as source for excitation light and a high numerical aperture oil immersion objective (Zeiss Plan-Neofluar, 100×/1.30) [19]–[20]. The system was equipped with an avalanche photodiode (APD, SPCM-AQR-14) as a spectrally integrating detector to record fluorescence intensity. Spectra were obtained by a spectrograph (Princeton Instruments Acton Spectra Pro 300i, 300 grid grating) coupled to a thermo-electric cooled CCD-camera (Roper Scientific). Lifetime decays were recorded using a time-correlated single-photon counting board (Timeharp 200, Picoquant) for data acquisition and the APD as a detector. Fluorescence intensity images were obtained by raster-scanning the sample and detecting emission intensity for every spot on the sampled area. Distinct spots were addressed to record the corresponding spectra. The pulsed 473 nm diode laser (Picoquant LDH-P-C470), operating at a repetition rate of 10 MHz served as source. The setup was equipped with a 480 nm long pass filter (Semrock Razor Edge LP02-473RU-25) to block back-scattered excitation light, with a 500 nm bandpass filter (Semrock BrightLine BL500/24) to detect GFP-fluorescence or with a 600 nm bandpass filter (Semrock BrightLine BL607/36) to detect autofluorescence. The lifetime data were recorded sequentially to invesitgate the emission lifetime of the GFP label and the autofluorescence background independently from each other. Acquisition time was 5 seconds per measurement. The pixel size is diffraction-limited and the distance between each measurement point was 250 nm. Processing of fluorescence intensity images was accomplished with the WSxM software (Nanotec Electronica) [36]. The intensity decay curves were deconvolved from the instrument response function (IRF) measured without the long pass filter and then fitted by a mono- or multi-exponential decay function.

The cell physiological experiments (cell wall expansion, BRI1-GFP and aquaporin lifetime measurements) were repeated at least 3 times using independent seedling samples.

### Probe preparation and transmission electron microscopy (TEM)

Seedlings were incubated in water or 10 nM brassinolide solution for 30 min at room temperature. Thereafter, root tips were high-pressure frozen and freeze-substituted in acetone containing 2.5% osmium tetroxide (60 h at −90°C, 8 h at −60°C and 8 h −35°C) before transfer for 60 min to 0°C [37]. Then, the root tips were washed five times with acetone followed by infiltration with 10% (4 h), 25% (12 h), 50% (12 h), 75% (12 h) and 100% (2 times for 15 h, each) epoxy resin. The resin was allowed to polymerise for 2 days at 60°C. Ultrathin sections of the root tip samples were stained with 3% uranyl acetate in ethanol and lead citrate and viewed in a LEO 906 transmission electron microscope [37]. 72 walls derived from two independent, mock-treated BRI1-GFP expressing seedlings and 72 walls derived from two independent, BL-treated BRI1-GFP expressing seedlings were measured.

### Data analysis

The standard deviations in the Gaussian fits of the intensity profiles, as represented by the error bars, reflect the accuracy of the fit and rest on the different signal-to-noise ratio of the measurements. The FWHM-based and TEM-based wall measurements of cells before/without and after treatment with BL were performed as indicated in the Tables S 2, S3, S4, S5, S6 and S7. For statistical evaluation, the data were analysed by one-sided t-test. The number of independent measurements (n), the p-values and the number of independent seedlings are given in the legends of the Tables S2, S3, S4, S5, S6 and S7 and in Results.

The lifetime values derived from mono- or multi-exponential decay fittings. The error bars were calculated according to [31] taking into account the number of photons and the time resolution of the measurements. The BRI1-GFP and aquaporin-GFP fluorescence lifetime measurements were repeated at least three times using individual cells from at least three independent seedlings and representative results are shown. The results of the additional experiments are presented in the Figures S2 and S3.

## Supporting Information

Figure S1 Reference fluorescence spectrum and lifetime decay rate of purified GFP. (A) Fluorescence spectrum of GFP at a concentration of 10−5 M after pulse excitation with light of 473 nm. The spectrum shows a peak at 510 nm and a shoulder at 540 nm. (B) Fluorescence decay rate of GFP at a concentration of 10−7 M in 20 mM TRIS (pH = 6.8) after pulse excitation with light of 473 nm. The decay shows a mono-exponential function. The residuals indicate the deviation between the measured and the model decay function. In a good fit the residuals are distributed symmetrically around 0. IRF, instrument response function.
(0.07 MB PDF)

Figure S2 BL induces changes in the BRI1-GFP fluorescence lifetime in plant cells. (A–B) Fluorescence lifetimes of BRI1-GFP in 4.0 µm plasmalemma-cell wall sections of two hypocotyl cells from two independent seedlings (A, B) before (black squares) and 10 (red squares), 20 (blue squares) and 30 min (green squares) after addition of 25 nM BL.
(0.08 MB PDF)

Figure S3 BL-induced cell wall expansion and change in BRI1-GFP fluorescence lifetime require a functional intracellular trafficking system. (A–C) FWHM values of GFP intensity profiles (left) and fluorescence lifetime decays (right) recorded over plasmalemmata-cell wall sections of three hypocotyl cells (A, B, C) from three independent, BRI1-GFP expressing Arabidopsis seedlings in the presence of 50 µM BFA before (black) and 30 min (green) after application of 25 nm BL.
(0.15 MB PDF)

Table S1 Fitting parameters of lifetime decay traces.
(0.17 MB XLS)

Table S2 FWHM changes in the widening of the plasmalemma-bound GFP fluorescence signal in BRI1-GFP expressing hypocotyl and root cells before (0 min) and 30 min after application of 10 nM BL (30 min.).
(0.01 MB PDF)

Table S3 FWHM changes in the expansion of the cell wall autofluorescence in BRI1-GFP expressing hypocotyl and root cells before (0 min) and 30 min after application of 10 nM BL (30 min).
(0.01 MB PDF)

Table S4 FWHM changes in the expansion of Calcofluor-stained cell walls in BRI1-GFP expressing hypocotyl and root cells before (0 min) and 30 min after application of 10 nM BL (30 min).
(0.01 MB PDF)

Table S5 Statistics of 72 periclinal cell wall width measurements, each, derived from ultrathin TEM sections of BRI-GFP expressing, high-pressure-frozen root cells, which were either mock-treated or treated for 30 min with 10 nM BL.
(0.01 MB PDF)

Table S6 FWHM changes in the widening of the plasmalemma-bound GFP fluorescence signal in aquaporin-GFP expressing hypocotyl and root cells before (0 min) and 30 min after application of 10 nM BL (30 min.).
(0.01 MB PDF)

Table S7 FWHM changes in the widening of the cell wall autofluorescence in wildtype hypocotyl and root cells before (0 min) and 30 min after application of 10 nM BL (30 min).
(0.01 MB PDF)

Text S1 Calculation of the width of plasmalemma-cell wall sections from apparent GFP fluorescence data (FWHM values).
(0.23 MB PDF)
