# Supplementary figures and images for: Novel Application of Fluorescence Lifetime and Fluorescence Microscopy Enables Quantitative Access to Subcellular Dynamics in Plant Cells
Source: PLoS One. 2009 May 27;4(5):e5716. doi: 10.1371/journal.pone.0005716 (PMC2683565; doi:10.1371/journal.pone.0005716)

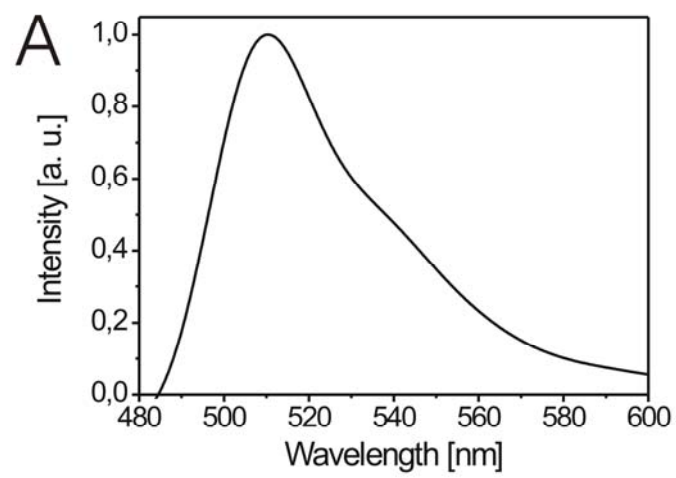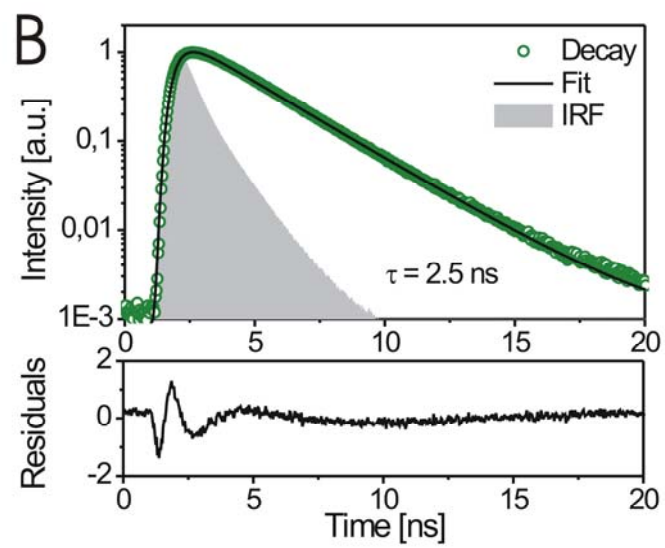

Supplement: Figure S1 — Reference fluorescence spectrum and lifetime decay rate of purified GFP. (A) Fluorescence spectrum of GFP at a concentration of 10−5 M after pulse excitation with light of 473 nm. The spectrum shows a peak at 510 nm and a shoulder at 540 nm. (B) Fluorescence decay rate of GFP at a concentration of 10−7 M in 20 mM TRIS (pH = 6.8) after pulse excitation with light of 473 nm. The decay shows a mono-exponential function. The residuals indicate the deviation between the measured and the model decay function. In a good fit the residuals are distributed symmetrically around 0. IRF, instrument response function. (0.07 MB PDF) [file pone.0005716.s001.pdf]

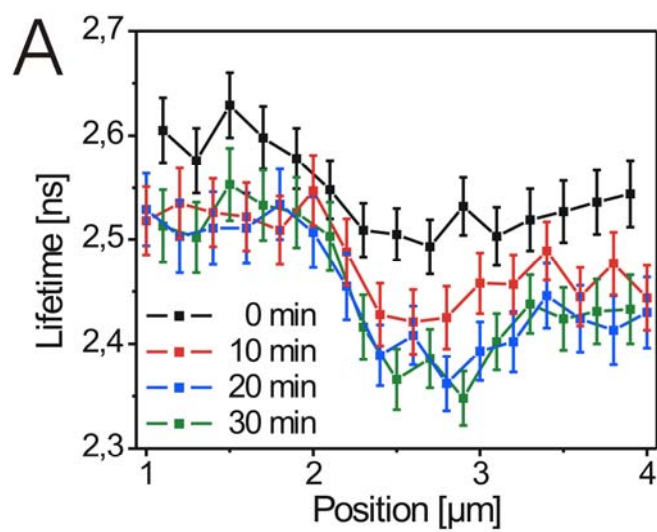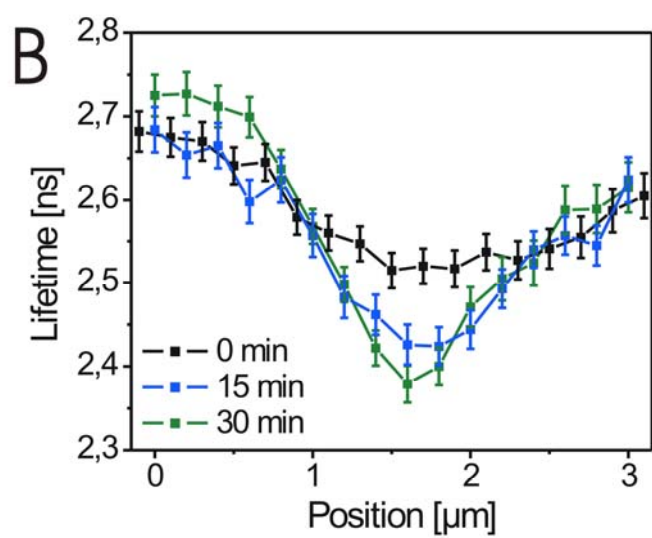

Supplement: Figure S2 — BL induces changes in the BRI1-GFP fluorescence lifetime in plant cells. (A–B) Fluorescence lifetimes of BRI1-GFP in 4.0 µm plasmalemma-cell wall sections of two hypocotyl cells from two independent seedlings (A, B) before (black squares) and 10 (red squares), 20 (blue squares) and 30 min (green squares) after addition of 25 nM BL. (0.08 MB PDF) [file pone.0005716.s002.pdf]

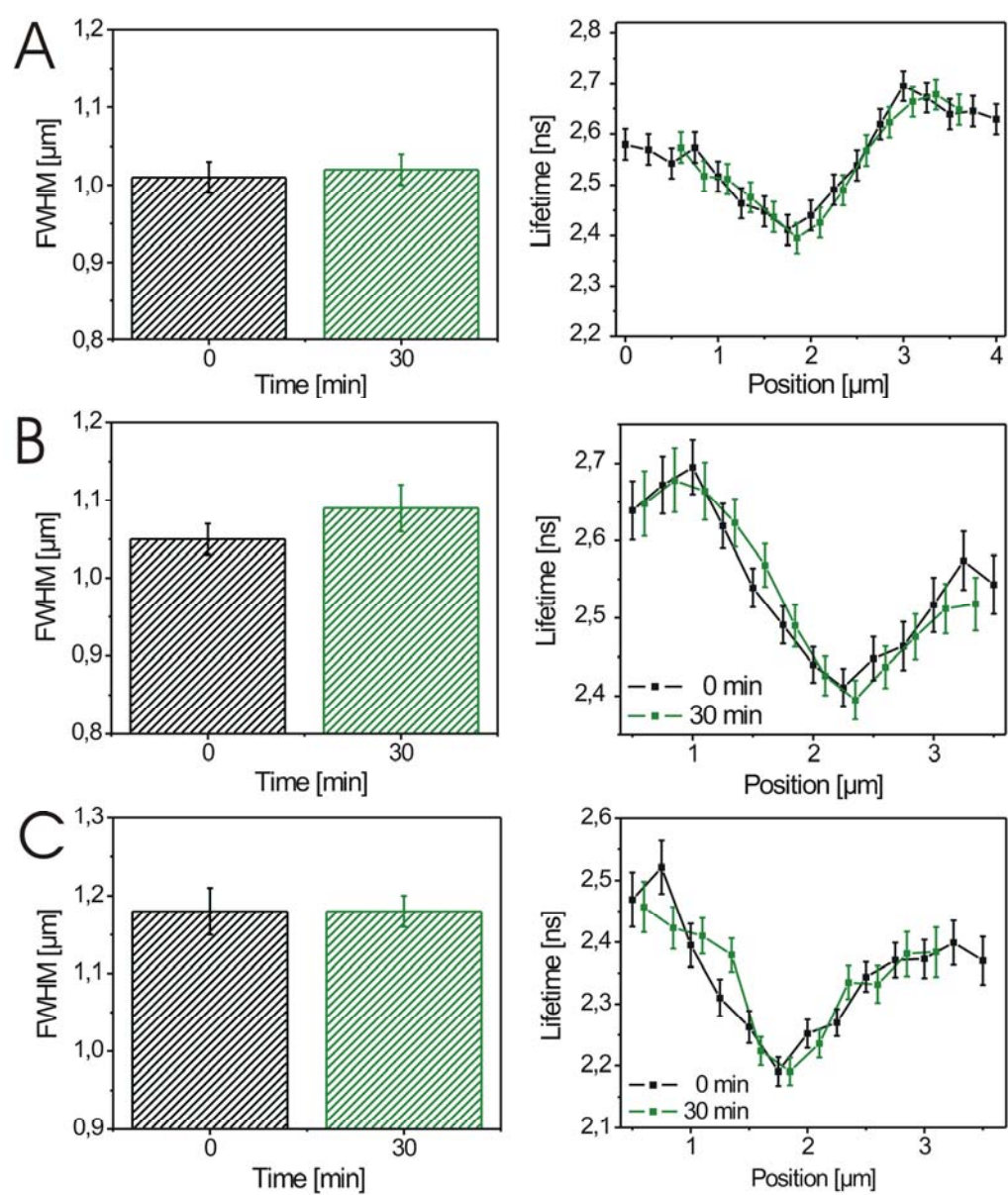

Supplement: Figure S3 — BL-induced cell wall expansion and change in BRI1-GFP fluorescence lifetime require a functional intracellular trafficking system. (A–C) FWHM values of GFP intensity profiles (left) and fluorescence lifetime decays (right) recorded over plasmalemmata-cell wall sections of three hypocotyl cells (A, B, C) from three independent, BRI1-GFP expressing Arabidopsis seedlings in the presence of 50 µM BFA before (black) and 30 min (green) after application of 25 nm BL. (0.15 MB PDF) [file pone.0005716.s003.pdf]
