# Supplementary material for: Novel Application of Fluorescence Lifetime and Fluorescence Microscopy Enables Quantitative Access to Subcellular Dynamics in Plant Cells
Source: PLoS One. 2009 May 27;4(5):e5716. doi: 10.1371/journal.pone.0005716 (PMC2683565; doi:10.1371/journal.pone.0005716)
Supplement: Table S2 — FWHM changes in the widening of the plasmalemma-bound GFP fluorescence signal in BRI1-GFP expressing hypocotyl and root cells before (0 min) and 30 min after application of 10 nM BL (30 min.). (0.01 MB PDF) [file pone.0005716.s005.pdf]

Suppl. Table 2. FWHM changes in the widening of the plasmalemma-bound GFP fluorescence signal in BRI1-GFP expressing hypocotyl and root cells before (0 min) and 30 min after application of 10 nM BL (30 min). The statistical analysis revealed an expansion of the cell wall by 34 ± 22 % (n = 31, p = 0,0003). The measurements were performed on 31 independent cells derived from 5 independent seedlings.

**FWHM [µm]**	**FWHM [µm]**	**expansion [%]**
**0 min**	**30 min**	
0,95	1,26	33
1,09	1,60	47
1,05	1,20	14
1,35	1,58	17
1,25	1,35	8
0,93	1,01	9
0,85	1,51	78
0,84	1,18	40
0,87	0,98	13
1,43	1,98	38
1,63	2,84	74
1,40	1,60	14
1,26	1,50	19
0,80	1,20	50
1,44	1,59	10
1,37	2,32	69
1,01	1,25	24
0,79	1,05	33
1,13	1,88	66
0,84	1,07	27
0,79	1,24	57
0,63	0,85	35
0,83	1,31	58
1,01	1,68	66
0,94	1,46	55
0,92	1,19	29
2,07	2,18	5
1,40	1,70	21
1,85	2,15	16
1,36	1,48	9
1,56	1,93	24
**mean**		**34**
**standard deviation**		**22**
