# Supplementary material for: Novel Application of Fluorescence Lifetime and Fluorescence Microscopy Enables Quantitative Access to Subcellular Dynamics in Plant Cells
Source: PLoS One. 2009 May 27;4(5):e5716. doi: 10.1371/journal.pone.0005716 (PMC2683565; doi:10.1371/journal.pone.0005716)
Supplement: Table S3 — FWHM changes in the expansion of the cell wall autofluorescence in BRI1-GFP expressing hypocotyl and root cells before (0 min) and 30 min after application of 10 nM BL (30 min). (0.01 MB PDF) [file pone.0005716.s006.pdf]

Suppl. Table 3 FWHM changes in the expansion of the cell wall autofluorescence in BRI1-GFP expressing hypocotyl and root cells before (0 min) and 30 min after application of 10 nM BL (30 min). The statistical analysis revealed an expansion of the cell wall by 41 ± 24 % (n = 29, p = 0,0145). The measurements were performed on 29 independent cells derived from 4 independent seedlings.

**FWHM [µm]**	**FWHM [µm]**	**Expansion [%]**
**0 min**	**30 min**	
1,06	1,67	58
1,05	1,35	29
0,96	1,26	31
0,85	0,97	14
2,05	2,46	20
0,98	1,25	28
0,52	0,63	21
0,38	0,50	32
0,33	0,48	45
0,30	0,59	97
0,31	0,46	48
0,80	0,95	19
0,55	0,96	75
1,06	1,54	45
0,68	1,04	53
1,25	1,48	18
1,61	1,86	16
1,45	1,91	32
1,50	2,23	49
1,47	2,86	95
0,50	0,80	60
1,13	1,79	58
1,81	2,34	29
1,94	2,73	41
1,70	1,88	11
1,02	1,83	79
1,13	1,36	20
1,44	2,04	42
2,93	3,28	12
**mean**		**41**
**standard deviation**		**24**
