# Supplementary material for: Novel Application of Fluorescence Lifetime and Fluorescence Microscopy Enables Quantitative Access to Subcellular Dynamics in Plant Cells
Source: PLoS One. 2009 May 27;4(5):e5716. doi: 10.1371/journal.pone.0005716 (PMC2683565; doi:10.1371/journal.pone.0005716)
Supplement: Table S5 — Statistics of 72 periclinal cell wall width measurements, each, derived from ultrathin TEM sections of BRI-GFP expressing, high-pressure-frozen root cells, which were either mock-treated or treated for 30 min with 10 nM BL. (0.01 MB PDF) [file pone.0005716.s008.pdf]

Suppl. Table 5 Statistics of 72 periclinal cell wall width measurements, each, derived from ultrathin TEM sections of BRI-GFP expressing, high-pressure-frozen root cells, which were either mock-treated or treated for 30 min with 10 nM BL. The statistical analysis revealed a cell wall width of 62,5 ± 10,8 nm for the mock-treated and 64,9 ± 10,1 nm for BL-treated seedlings. There is a weak difference with a p-value of 0,0845.

CWW [nm] mock-treated	CWW [nm] BL-treated
65,5	49,8
72,6	56,4
55,1	51,2
64,5	71,3
50,3	53,2
81,1	55,5
83,4	49,3
86,7	66,8
86,8	60,9
60,3	64,2
56,2	66,3
47,3	60,3
50,1	68,4
53,7	67,9
48,9	59,2
55,0	65,4
64,2	63,7
80,0	67,2
71,8	60,4
45,0	52,9
61,0	59,0
56,8	53,2
62,5	63,3
52,4	61,7
58,8	58,7
50,7	61,7
60,7	62,1
64,6	63,0
68,3	64,8
56,5	77,7
57,0	73,6
63,8	71,6
63,1	63,4

	55,3	65,0
	65,3	73,3
	61,5	67,2
	58,6	50,5
	58,2	55,9
	50,6	74,1
	68,0	63,5
	49,0	53,0
	60,5	66,4
	56,7	59,1
	78,1	62,8
	48,1	63,1
	35,7	75,7
	68,1	65,0
	60,7	60,1
	60,4	75,5
	58,8	78,6
	54,0	81,9
	55,1	85,2
	57,2	94,2
	51,8	87,8
	50,6	48,9
	74,1	69,3
	67,0	48,8
	80,3	56,1
	67,3	56,2
	71,1	53,6
	82,2	59,6
	87,9	66,7
	79,3	67,6
	68,7	63,8
	63,3	63,5
	66,1	63,7
	56,8	74,4
	68,8	75,8
	56,9	91,7
	58,9	62,4
	66,8	85,7
	64,1	60,2
**mean**	**62,5**	**64,9**
**standard deviation**	**10,8**	**10,1**
