# Supplementary material for: Novel Application of Fluorescence Lifetime and Fluorescence Microscopy Enables Quantitative Access to Subcellular Dynamics in Plant Cells
Source: PLoS One. 2009 May 27;4(5):e5716. doi: 10.1371/journal.pone.0005716 (PMC2683565; doi:10.1371/journal.pone.0005716)
Supplement: Table S6 — FWHM changes in the widening of the plasmalemma-bound GFP fluorescence signal in aquaporin-GFP expressing hypocotyl and root cells before (0 min) and 30 min after application of 10 nM BL (30 min.). (0.01 MB PDF) [file pone.0005716.s009.pdf]

Suppl. Table 6 FWHM changes in the widening of the plasmalemma-bound GFP fluorescence signal in aquaporin-GFP expressing hypocotyl and root cells before (0 min) and 30 min after application of 10 nM BL (30 min.). The statistical analysis revealed a change of the cell wall width by 10 ± 8 % (n = 12, p = 0,187). The measurements were performed on 12 independent cells derived from 3 independent seedlings.

**FWHM [µm]**	**FWHM [µm]**	**Expansion [%]**
**0 min**	**30 min**	
0,71	0,75	6
0,78	0,92	18
0,92	0,97	5
0,81	0,89	10
0,90	0,99	10
0,77	0,80	4
1,45	1,47	1
1,22	1,24	2
1,11	1,20	8
1,04	1,36	31
1,07	1,19	11
1,47	1,67	14
**mean**		**10**
**standard deviation**		**8**
