# Supplementary material for: Novel Application of Fluorescence Lifetime and Fluorescence Microscopy Enables Quantitative Access to Subcellular Dynamics in Plant Cells
Source: PLoS One. 2009 May 27;4(5):e5716. doi: 10.1371/journal.pone.0005716 (PMC2683565; doi:10.1371/journal.pone.0005716)
Supplement: Table S7 — FWHM changes in the widening of the cell wall autofluorescence in wildtype hypocotyl and root cells before (0 min) and 30 min after application of 10 nM BL (30 min). (0.01 MB PDF) [file pone.0005716.s010.pdf]

Suppl. Table 7 FWHM changes in the expansion of the cell wall autofluorescence in wildtype hypocotyl and root cells before (0 min) and 30 min after application of 10 nM BL (30 min.). The statistical analysis revealed a change of the cell wall width by 18 ± 16 % (n = 11, p = 0,2607). The measurements were performed on 11 independent cells derived from 3 independent seedlings.

**FWHM [µm]**	**FWHM [µm]**	**Expansion [%]**
**0 min**	**30 min**	
0,73	0,84	15
0,95	1,12	18
1,32	1,96	48
1,33	1,54	16
0,66	0,98	48
0,93	0,95	2
3,10	3,60	16
2,00	2,27	14
2,96	3,33	13
1,49	1,52	2
2,50	2,70	8
**mean**		**18**
**standard deviation**		**16**
