# Supplementary material for: Novel Application of Fluorescence Lifetime and Fluorescence Microscopy Enables Quantitative Access to Subcellular Dynamics in Plant Cells
Source: PLoS One. 2009 May 27;4(5):e5716. doi: 10.1371/journal.pone.0005716 (PMC2683565; doi:10.1371/journal.pone.0005716)
Supplement: Text S1 — Calculation of the width of plasmalemma-cell wall sections from apparent GFP fluorescence data (FWHM values). (0.23 MB PDF) [file pone.0005716.s011.pdf]

Supplementary Material

Calculation of the width of the plasmalemmata-cell wall section from apparent GFP fluorescence data (ω (= FWHM) values).

The apparent ω of a plasmalemmata-cell wall section in a 2-dimensional fluorescence-intensity image of a GFP-labeled plasmalemma-located protein in plant cells is composed of two plasmalemmata, which are actually labeled by GFP fluorescence, and the cell wall (Fig. SM 1). Thus, the GFP fluorescence and cell wall autofluorescence overlap spatially and spectroscopically.

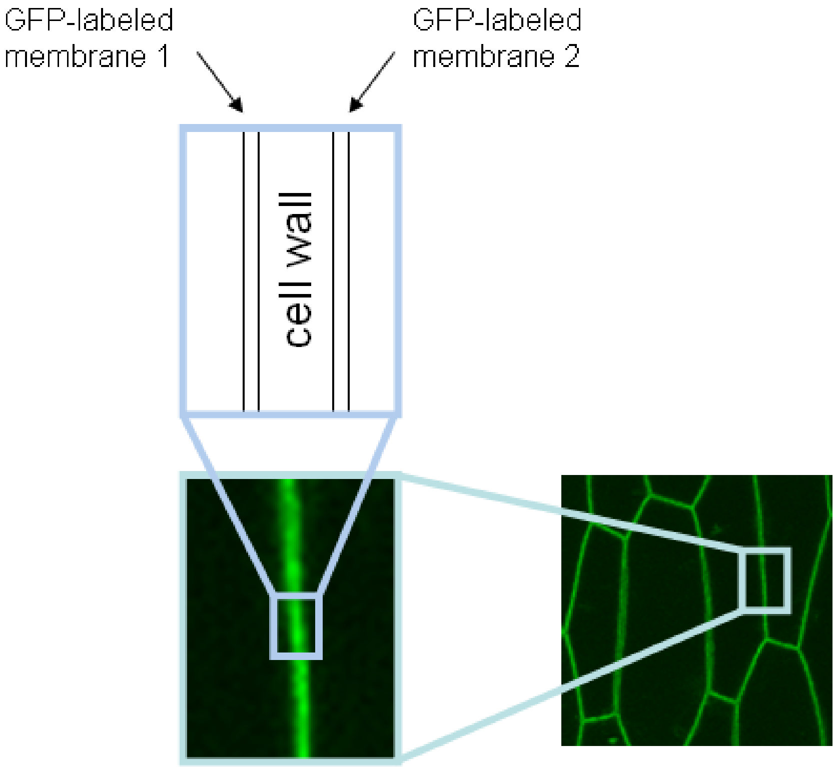

Figure SM 1. CSSM images of hypocotyl cells expressing BRI1-GFP and the corresponding scheme.

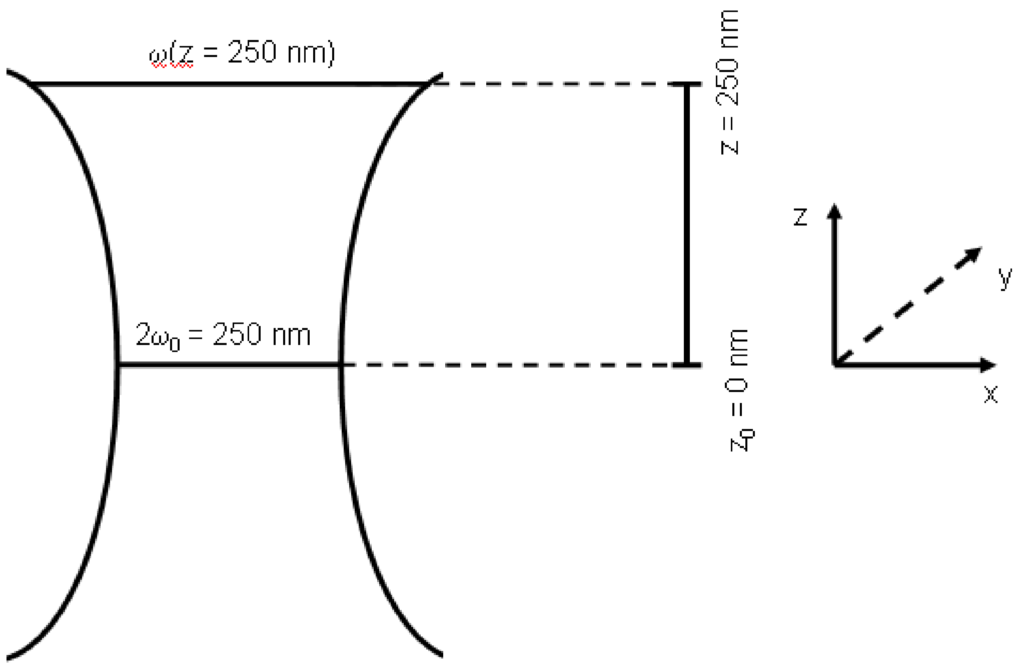

Figure SM 2. Dimensions of the focus in a laser-scanning confocal microscope with $\lambda_{Laser} \approx$ 500 nm. ω_0 = 250 nm / 2·NA = 125 nm (NA: Numerische Apertur≈ 1)

The calculation of the apparent thickness in the confocal laser microscope is based on the dimension of its focus (Fig. SM 2), the real thickness of a GFP-labeled plasmamembrane [10 nm; Strasburger – Lehrbuch der Botanik (2008) 36. Auflage] of the cell wall (100 nm; see Fig. 3 and 7 in the manuscript) and the fact that the membrane as well as the cell wall are not limited to the xy-layer, but also expand in z-direction. The focus gets broader with increasing z-distance. Thus, ω(z) with z = 250 nm can be calculated as follows [Jahns J, (2001) Photonik. Oldenbourg Verlag].

$$\omega(z) = \omega_0 \left[1 + \left(\frac{z}{z_0} \right)^2 \right]^{1/2}$$

$$z_0 = \frac{\pi \cdot \omega_0^2}{\lambda}$$

The calculation with the assumed parameters results in

$z_0 \approx 98\text{nm}$

$$\omega(z = 250\text{nm}) = 125\text{nm}\left[1 + \left(\frac{250\text{nm}}{98\text{nm}}\right)^2\right]^{½} = 342.5\text{nm} = \omega_{PSF}$$

with PSF being the microscope´s point spread function.

The convolution results in:

$$\omega_{con} = \sqrt{\omega_{ideal} + \omega_{PSF}} = \sqrt{10\text{nm}^2 + 342.5\text{nm}^2} = 342.65\text{nm}$$

For two Membranes with an interjacent cell wall in:

$$\omega_{final} = \omega_{con} + \text{cell wall thickness} = 342.65\text{nm} + 100\text{nm} = 442.65\text{nm}$$

Thus, a plasmalemmata-cell wall section of 120 nm wide appears in the confocal microscope with a size of **442.65** nm.

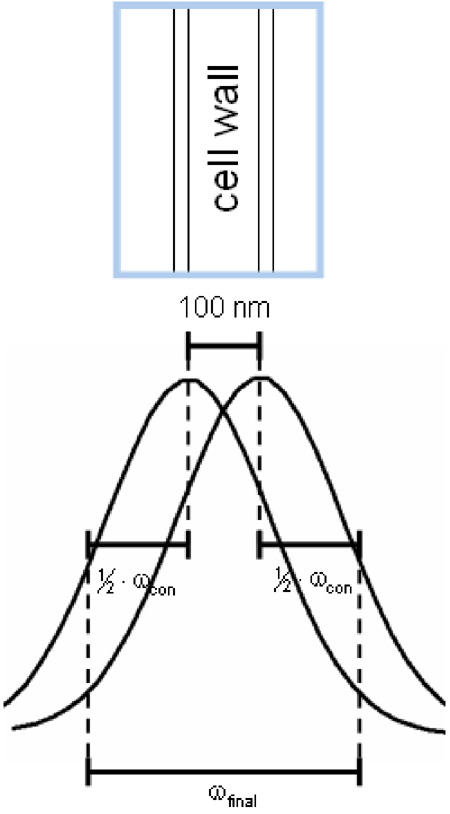

Figure SM 3. Calculation of ω_{final}.

However, the apparent ω_{final} changes, when the plasmalemmata-cell wall section is slanted by the angle (α) of 60° in the focus (Fig. SM4), and results in $\omega_{slanted}$:

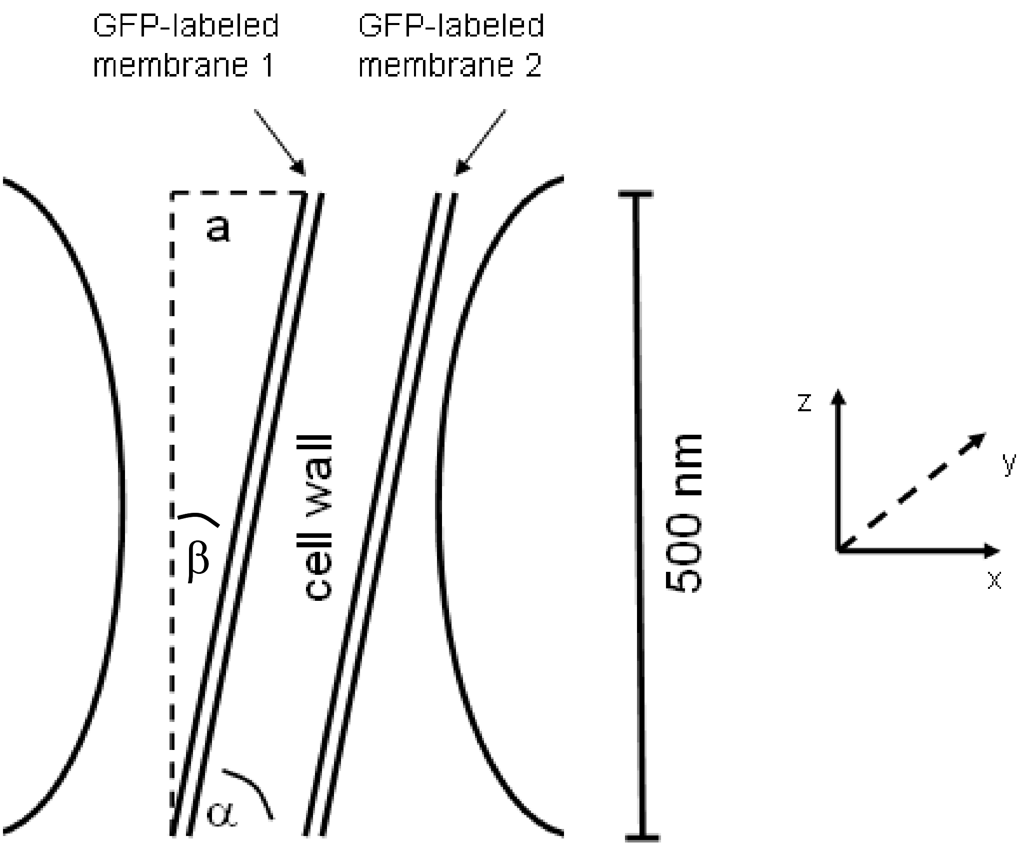

Figure SM 4. Dimensions of the microscopic focus, when the plasmalemmata-cell wall section is slanted by the angle (β) of 30°.

With the assumed parameters, the distance a can be calculated with α = 60° to:

$$\tan\beta = \tan(90° - \alpha) = \frac{\text{opposite leg}}{\text{adjacent leg}} = \frac{a}{500\text{nm}}$$

$$\text{and: } a = 0.5774 \cdot 500\text{nm} = 288.7\text{nm}$$

And the summation results in:

$$\omega_{slanted} = \omega_{final} + a = 442.65\text{nm} + 288.7\text{nm} = 731.35\text{nm}$$

Thus, a plasmalemmata-cell wall section of 120 nm wide appears in the confocal microscope with a size of **731.35** nm when the object is slanted by β = 30°.
